# Molecular and Cellular Mechanisms Underlying the Initiation and Progression of Alport Glomerular Pathology

**DOI:** 10.3389/fmed.2022.846152

**Published:** 2022-02-09

**Authors:** Dominic Cosgrove, Jacob Madison

**Affiliations:** Boys Town National Research Hospital, Omaha, NE, United States

**Keywords:** glomerulus, glomerular basement membrane, podocyte, mesangial cell, pathology

## Abstract

Alport syndrome results from a myriad of variants in the COL4A3, COL4A4, or COL4A5 genes that encode type IV (basement membrane) collagens. Unlike type IV collagen α1(IV)_2_α2(IV)_1_ heterotrimers, which are ubiquitous in basement membranes, α3/α4/α5 have a limited tissue distribution. The absence of these basement membrane networks causes pathologies in some, but not all these tissues. Primarily the kidney glomerulus, the stria vascularis of the inner ear, the lens, and the retina as well as a rare link with aortic aneurisms. Defects in the glomerular basement membranes results in delayed onset and progressive focal segmental glomerulosclerosis ultimately requiring the patient to undergo dialysis and if accessible, kidney transplant. The lifespan of patients with Alport syndrome is ultimately significantly shortened. This review addresses the consequences of the altered glomerular basement membrane composition in Alport syndrome with specific emphasis on the mechanisms underlying initiation and progression of glomerular pathology.

## Introduction

In terms of rare diseases, Alport syndrome is relatively common with recent genetic evidence suggesting a frequency approaching 1 in 2,000 people (1). Most affected individuals (80-85%) harbor variants in the X-linked COL4A5 gene, and thus are hemizygous with mosaicism in female carriers associated with a milder form of the disease who pass the variants to half of their sons. The remaining cases are caused by variants in COL4A3 and COL4A4 genes and are inherited in an autosomal recessive manner (2, 3). There is a strong genotype/phenotype association with the severity of kidney disease, with about 40% of the cases caused by Gly-X-Y missense variants, which result in a milder form of the disease and more severe variants (nonsense variants deletions, etc.) accounting for most cases (4). In most, but not all cases, the variants lead to the absence of all three type IV collagen chains in the glomerular basement membrane (GBM). This change in basement membrane composition results in a GBM that is notably thinner and contains fewer interchain disulfide crosslinks which weakens the elastic integrity of the glomerular capillary tufts making them susceptible to the mechanical forces of normal capillary blood flow (5). Consistent with this, glomeruli from Col4A3-/- Alport mice have a 30% reduction in Young's modulus, a measure of biophysical stiffness (6). When the capillary tufts are subjected to excessive forces under hypertensive conditions, the rate of disease progression is accelerated in the mouse (7). It was recently shown using intravital microscopy that the glomerular capillaries in Alport mice have a significantly enlarged diameter compared to wild type mice likely owing to the enhanced elasticity of the thinner and less crosslinked GBM collagen network (8). This may contribute to why ACE inhibitors, which reduce intraglomerular pressure, slow the progression of Alport kidney disease in both mice and human (9–11). A recent mass-spectroscopy-based proteomics study demonstrated aan overall reduction in basement membrane proteins in glomerular matrix from Alprot mice relative to wild type mice with an overall increase in interstitial matrix proteins (12). These changes are likely to contribute to the biomechanical prpoperties of the GBM in Alport syndrome. Of note, there are some variants that change the biochemical properties of the GBM without eliminating α3/α4/α5 protomers. For example, a variant that causes in frame skipping of exon 30 results in a GBM with normal type IV collagen composition (albeit less α3/α4/α5 networks than wild type) results in a robust Alport kidney phenotype in both mice and men (13). This underscores the importance of the structural integrity of the GBM in maintaining normal GBM function and supports the idea that biomechanical strain is a major contributor to disease onset.

The mature glomerular basement membrane is two separate networks of type IV collagen; a subendothelial network comprised of type IV collagen α1_2_/α2 protomers and a thicker subepithelial network comprised of type IV collagen α3/α4/α5 protomers (14). These networks contribute to both the structural integrity and the assembly of the GBM (15, 16). It has been suggested based on super resolution analysis of GBM protein architecture that the type IV collagen α1_2_/α2 network might be close enough to activate podocyte collagen receptors discoidin domain receptor 1 (DDR1) and integrin α2β1 while the α3/α4/α5 network in wild type mice is central to the GBM and thus too distant to engage collagen receptors on podocytes (17). Double knockouts of COL4A3 and either DDR1 or integrin α2 show attenuated kidney disease progression, implicating these receptors as contributors to the pathobiology of Alport glomerular disease (18, 19). Definitive proof of collagen receptor activation in Alport podocytes is lacking, however.

## Alport Glomerular Disease Onset

Even though the GBM type IV collagen network is congenitally structurally different in Alport syndrome, the GBM functions to prevent proteinuria and maintain a healthy glomerular filtration rate for several weeks in mice and several years in humans. If this functional competence could be maintained through therapeutic intervention, end stage kidney disease (ESRD) can be delayed, and lifespan increased. After proteinuria is established the current standard of care, angiotensin converting enzyme inhibitors (ACEi), have reduced efficacy (10, 11). This is also true in the mouse models (9). Thus, the driver(s) of glomerular disease onset should reveal therapeutic target(s) with maximum efficacy for blocking or slowing the Alport kidney disease progression.

One of the earliest events documented to date is significantly elevated expression of endothelin-1 (ET-1) in the endothelial cells of Alport glomeruli (20). This is observed as early as 2 weeks in the 129 Sv ARAS model and 5 weeks in the Bl/6 XLAS model; before proteinuria is observed and before notable ultrastructural defects in the GBM are observed. Expression of ET-1 is further elevated when mice are made hypertensive, suggesting that the induction of ET-1 is a consequence of elevated biomechanical strain on the glomerular capillary tufts. One consequence of elevated ET-1 expression is the activation of endothelin A receptors (ET_A_Rs) on mesangial cells. Activated ET_A_Rs lead to downstream activation of CDC42 resulting in the formation of mesangial filopodia. The filopodia progressively invade the sub-endothelial aspect of the glomerular basement membrane and deposit mesangial matrix proteins including laminin 211 in the GBM (21). Filopodial invasion was validated by 3-dimensional electron microscopy (22). In this same work, podocyte process invation of the GBM was also reported. Laminin 211 has been shown to activate focal adhesion kinase(FAK) on glomerular podocytes resulting in NF-kappaB activation which results in elevated expression of pro-inflammatory cytokines as well as matrix metalloproteinases (23). Blocking FAK or ET_A_Rs with small molecule inhibitors ameliorates Alport GBM disease (20, 23, 24). Laminin 211 is deposited in the GBM in Alport dog models as well as humans (20, 25) confirming this process as a relevant pathological mediator in patients with Alport syndrome. In addition to aberrant cell signaling, laminin 211 in the GBM contributes to the GBM permeability defects (26), suggesting its progressive accumulation in the GBM is relevant to the progressive increase in proteinuria. Thus, disease onset involves three glomerular cell types: cross-talk between the glomerular endothelial cells and mesangial cells resulting in the deposition of mesangial ECM in the GBM, which causes podocyte injury.

**Figure 1:**
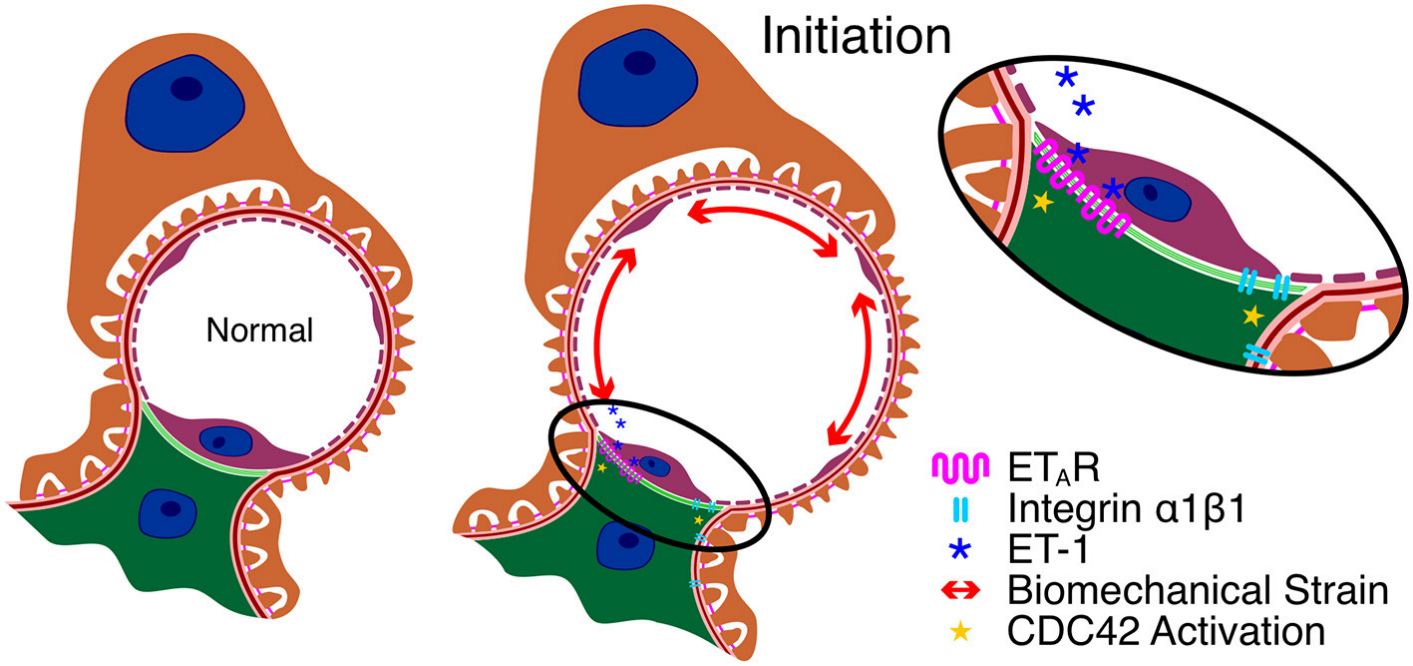
Key aspects of Alport glomerular disease initiation. Due to the change in basement membrane composition, the GBM in Alport syndrome is thinner and more elastic, resulting in increased capillary diameter and biomechanical strain on the cells that adhere to the capillary. This results in elevated expression of ET-1 in the endothelial cells which activates ET_A_Rs on mesangial cells resulting in CDC42 activation. Blockade of integrin α1β1 on mesangial cells prevents ET_A_R-mediated CDC42 activation and thus prevents initiation through an unknown mechanism.

Over 20 years ago, a connection between integrin α1β1 and the deposition of laminin 211 in Alport GBM was described where deletion of *ITGA1* significantly slowed the rate of laminin 211 deposition in the GBM and increased lifespan by 50% (27). Since then, we noted that α1β1 integrin in glomerular mesangial cells is required for lipopolysaccharide-mediated activation of CDC42 and the formation of mesangial filopodia (21). Thus, there appears to be a convergence between blocking integrin α1β1 receptors and blocking ET_A_Rs in the activation pathway for CDC42 in glomerular mesangial cells and thus α1 integrin itself may be a relevant target for blocking events related to glomerular disease initiation in Alport syndrome. Key aspects of Alport glomerular disease initiation are summarized in Figure 1.

## Alport Glomerular Disease Progression

Once the mesangial filopodia begin to invade the subendothelial space of the glomerular capillaries, we begin to observe the deposition of mesangial matrix proteins in the GBM. This includes laminin 211, which has been implicated in directly injuring podocytes (23). While podocyte injury by laminin α2 was shown to be mediated by FAK activation leading to NFkappaB activation and the induction of MMPs and pro-inflammatory cytokines, the specific receptors on podocytes that mediate FAK activation have not yet been identified. Collagen-mediated podocyte damage was shown to be the result of DDR1 and integrin α2β1 activation (18, 19). The downstream effector functions of this co-receptor signaling includes both previously recognized Alport glomerular disease mediators as well as several genes that have been implicated in podocyte injury and/or CKD in other disease models but not yet implicated in Alport glomerular pathogenesis. Previous work showed that double knockouts of COL4A3 and either DDR1 or integrin α2β1 resulted in attenuated kidney disease progression in the autosomal Alport mouse model, clearly implicating aberrant DDR1 and/or α2β1 signaling in Alport glomerular pathogenesis (18, 19).

One of the earliest cytokines implicated in Alport kidney and glomerular pathogenesis was TGF-β1 (28, 29). In the glomerulus, TGF-β1 blockade prevented GBM dysmorphology but did not improve lifespan (27) in the interstitium, TGF-β1 blockade prevented the genesis of interstitial myofibroblasts and interstitial fibrosis (30). TGF-β1 signaling has been directly implicated in studies using STAT3 inhibitors which slowed the progression of Alport kidney pathogenesis in a mouse model (31). Blockade of the Activin type II receptor, a member of the TGF-β receptor family, attenuated Alport glomerular and interstitial disease progression as well (32). BMP-7 is an inhibitor of the TGF-β signaling cascade that induces fibrosis. It has been shown in animal models, including an Alport mouse model, that recombinant BMP-7 ameliorates kidney disease (33). Along these same lines, deletion of the natural BMP-7 antagonist uterine sensitization-associated gene 1 (USAG1) in an Alport mouse model ameliorated progression of glomerular and interstitial disease (34). TGF-β1 might be an early indicator of Alport kidney pathogenesis as both serum and urinary TGF-β1 were significantly higher in pre-proteinuric Alport patients compared to controls (35). Collectively this work makes a definitive connection between TGF-β and Alport kidney disease progression.

Cholesterol accumulates in the podocytes in models of focal segmental glomerulosclerosis (FSGS) as well as Alport syndrome (36). Treatment with cyclodextrins which promote cholesterol efflux ameliorates the progression of Alport kidney disease. These compounds cause significant hearing loss in humans (37) necessitating the identification of alternate means to stimulate cholesterol efflux. Recently small molecules have been identified that activate ATP-binding cassette transporter (ABCA1)-mediated cholesterol efflux via targeting Oxysterol Binding Protein Like 7 (OSBPL7) (38). This approach also ameliorated Alport kidney disease progression and increased lifespan by about 20%. Interestingly DDR1 activation is linked to lipotoxicity (39), which may partially explain why deletion of DDR1 in Alport mice slows kidney disease progression (18).

Capillary endothelial cell damage also contributes to Alport glomerular disease progression. It was shown that injection of amniotic fluid stem cells would slow the progression of Alport kidney disease (40). It was later shown that vesicles derived from these stem cells protect against VEGF-induced endothelial cell damage, showing that VEGF plays an important role in Alport glomerular pathology and that trapping of VEGF by these vesicles represents a viable targeted approach for slowing the disease progression (41).

**Figure 2:**
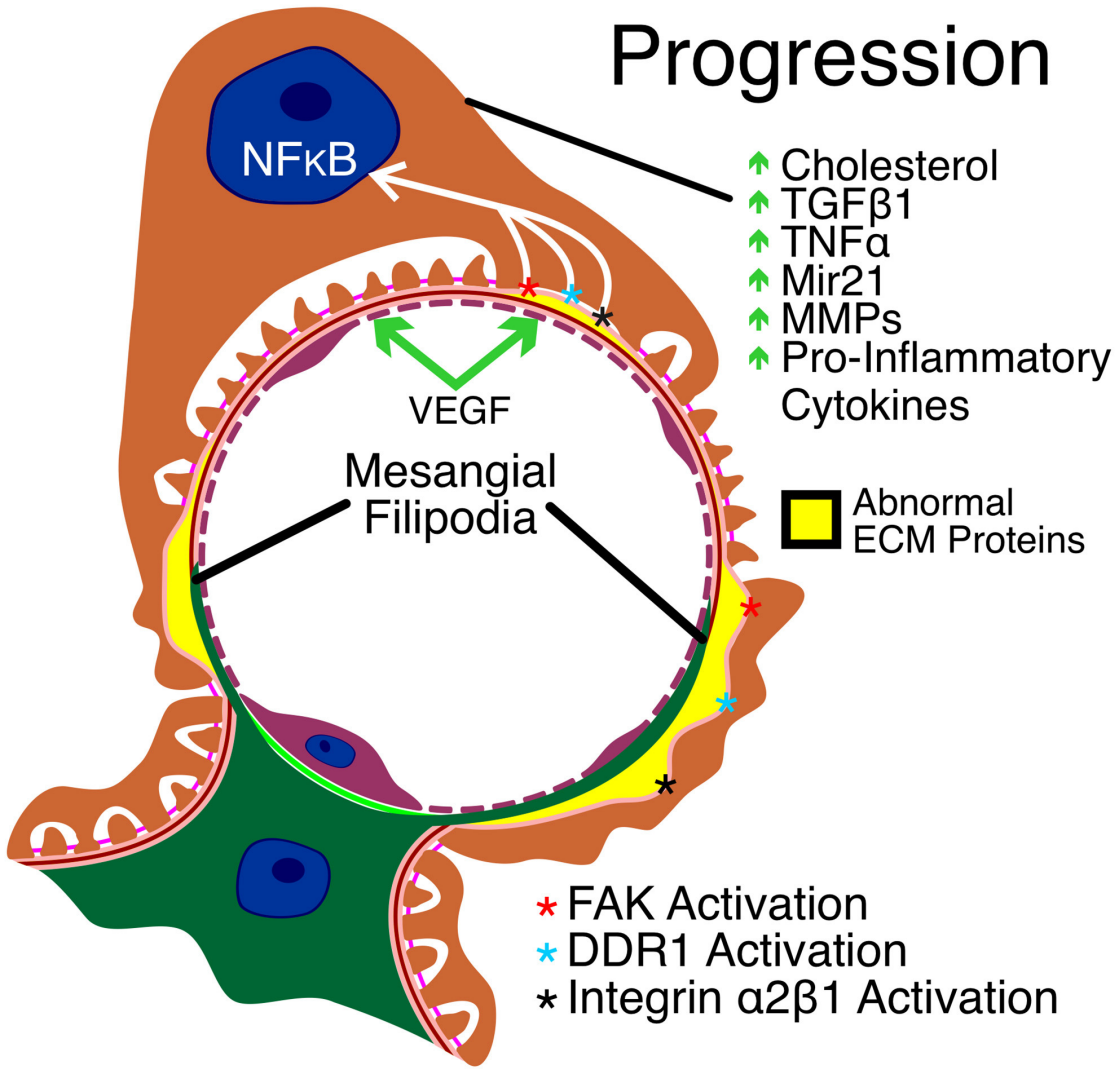
Key aspects of Alport glomerular disease progression. As a result of CDC42 activation, mesangial filopodia invade the space between the endothelial cells and the GBM. The abnormal ECM accumulates in the apical aspects of the GBM, contributing to the irregular thickening of the GBM (in yellow) and activating yet unknown laminin α2 receptors resulting in FAK activation and downstream NF-kappaB activation. In addition, collagen α1(III) in the GBM activates DDR1 and α2β1 receptors on podocytes leading to further podocyte injury. The resulting injury to podocytes in mediated by cholesterol retention and elevated expression of TGF-β1, TNF-α, mir21, MMPs and several pro-inflammatory cytokines. Endothelial cell injury is mediated by elevated VEGF.

Podocytopenia is observed in Alport syndrome. Tumor necrosis factor-α has been implicated in podocyte apoptosis that might contribute to reduced podocyte numbers (42). More recently it was shown that Alport podocytes during progression of the glomerular disease re-enter the cell cycle, passing the G1/S checkpoint which can lead to podocyte detachment, contributing to reduced podocyte numbers (43). Podocytopenia has been directly linked to glomerulosclerosis in models of FSGS, suggesting that TNF-α may be a prominent effector of glomerular disease progression in Alport syndrome (44). Key aspects of Alport glomerular disease progression are summarized in Figure 2.

## Clinical Trials: Where are We Now?

Many potential therapeutic targets have been inferred from pre-clinical studies of the mouse model, but never made it to clinical trials. These include key cell signaling targets including the BMP antagonist USAG1 (34), α1β1 integrin (27) α2β1 integrin (19), DDR1 (18), αvβ6 integrin (45), STAT3 (31), lysyl oxidase like-2 (46), focal adhesion kinase (23) and osteopontin (47). Other targets include epigenetic modification by histone deacetylases (48), and matrix metalloproteinases including MMP 12 (49). Recently the diabetes drug metformin was shown to ameliorate the severity of Alport kidney disease in the XLAS mouse model (50). While metformin had no effect on extending lifespan in the 129 Sv ARAS model it did moderately increase lifespan in the Bl/6 XLAS model. All of these approaches act on elements of disease progression, which likely accounts for their limited capacity to slow disease progression and increase lifespan.

Other pre-clinical studies have been conducted that attempt to replace the defective type IV collagen gene product with a fully functional one. Since these approaches actually aim to repair the GBM composition, they have the capacity to arrest glomerular disease initiation and thus provide maximum therapeutic benefit. The first attempt was using gene therapy to introduce a full-length type IV collagen α5 mRNA into dog podocytes by direct effusion of viral particles into the kidney artery. The work showed some promise but was ultimately unsuccessful (51). Heidet et al. (52) showed that introduction of the human α3(IV)/α4(IV) genes by way of transgenesis using a yeast artificial chromosome rescued the phenotype, albeit small defects in the GBM were observed. Related to this, Lin et al. (53) showed using a tamoxifen-inducible transgene, that introduction of full-length collagen α3(IV) would fully rescue when the transgene was induced at post-natal day 1, and to a lesser extent when introduced at post-natal day 21 (pre-proteinuric mice), providing proof of concept that gene therapy (or gene editing) may be a feasible approach for improving kidney function in Alport patients. Stem cell therapy in Alport mice has also been attempted using bone marrow-derived stem cells (54, 55) and stem cells from amniotic fluid (40) with very limited success.

Several therapeutic approaches are in either the planning or active stages of clinical trials in Alport patients. These include Bardoxylone methyl, a nuclear factor erythroid 2–related factor 2 (NRF2) agonist that regulates reduction/oxidation reactions and NF-kappa B activation, which successfully filed an NDA. Recent setbacks suggest that this approach might not meet FDA expectations. Concerns included a lack of pre-clinical work in animal models and a poorly understood increase in proteinuria that may reflect elevated intraglomerular pressure which itself could be pathologic. The endothelin A receptor antagonist (ET_A_R) atrasentan is currently in Phase 2 clinical trials for Alport syndrome. Of note, a similar ET_A_R antagonist, sitaxentan, improved kidney function in Alport mice, but increased lifespan by only 20% (20). Recruitment of Alport patients has begun for a planned trial using the anti-microRNA-21 Lademirsen, which was shown in Alport mice studies to improve kidney function and lifespan by 50% (56). Preliminary work in humans using sodium-glucose cotransporter-2 inhibitors showed some promise in Alport patients and other forms of CKD, however larger numbers will be needed to see if the data is indeed significant (57). Importantly, none of these therapeutic approaches has been shown in animal models to provide any benefit beyond the standard of care (ACEi or ARBs) except for metformin, and for this only in the 129 Sv ARAS model (50).

## Summary

The initiation and progression of Alport glomerular pathogenesis involves extensive cellular crosstalk between the endothelial cells, mesangial cells, and podocytes. Mesangial cell-derived ECM deposition in the GBM clearly plays an important role in aberrant podocyte cell signaling resulting in podocyte injury which is a major of glomerular disease progression. Given that Alport syndrome is the result of variants in COL4A3, COL4A4 or COL4A5 genes, the ultimate therapy would correct the variant(s) using a gene editing approach, replace the defective network using recombinant collagen α3/α4/α5 protomers, or introduce a correct copy of the defective gene to enough podocytes to be therapeutically beneficial. Such procedures would likely need to be administered before the onset of proteinuria. Until such therapies are available the best approach would be to delay the glomerular disease onset for as long as possible and then use approaches that slow progression additively or synergistically with the standard of care (ACEi or ARBs).

## Author Contributions

DC wrote and edited the manuscript. JM produced the figures and edited the manuscript. Both authors contributed to the article and approved the submitted version.

## Conflict of Interest

The authors declare that the research was conducted in the absence of any commercial or financial relationships that could be construed as a potential conflict of interest.

## Publisher's Note

All claims expressed in this article are solely those of the authors and do not necessarily represent those of their affiliated organizations, or those of the publisher, the editors and the reviewers. Any product that may be evaluated in this article, or claim that may be made by its manufacturer, is not guaranteed or endorsed by the publisher.
